# The Windham Case

**Published:** 1862-07

**Authors:** 


MEDICAL CRITIC

AND

PSYCHOLOGICAL

JOURNAL.

JULY, 1862.

### Art. I.—THE WINDHAM CASE.

By a Member of the Bar.

While the judicial proceedings in this case remained undetermined, we deemed it advisable to forbear expressing an opinion. Since the issue of the last number of this journal, the decision of the Lords Justices on the question of costs has terminated the litigation which the inquiry immediately involved. Their decision has done more,—it has afforded a conclusive answer to the unmeasured imputations indiscriminately heaped on those at whose instance the commission was issued. The whole proceeding is now an affair of the past, which we would willingly commit to oblivion, were it not that its consideration offers a series of disclosures without a parallel in the history of vice or folly, and invites to the discussion of questions provocative of criticism from every journalist interested in the public welfare.

All are familiar with the inquiry and its result.

It is now idle to speculate on the propriety of the verdict. It has been received and acted on as a fact. A jury of his countrymen have found that William Frederick Windham "was a person of sound mind; so as to be sufficient for the government of himself, his manners, his messuages, his lands, his tenements, his goods, and his chattels." We dismiss the gentleman from our consideration; and accepting as materials for argument the sworn and admitted testimonies of the several witnesses examined during the investigation, will essay a practical view of a practical question.

The petitioners asserted that the subject of their proposed inquiry was incompetent for the management of his person or property, and that he was so from unsoundness or deficiency of mind. They set forth evidence to show that the unsoundness or deficiency of mind, of which the management of property was the consummation, had been present from his youth, more or less manifest, as opportunity or occasion offered. To establish this proposition it became requisite to review the history of many years, and to trace Mr. Windham's progress from youth to manhood. The propriety of this course was obvious; nay more, it was a necessity. To those conversant with the intricate and anomalous phases which mental unsoundness not unfrequently presents, it may be unnecessary to detail reasons for so doing. To those who are less learned, we may premise that the issue raised by the petitioners involved the inquiry whether Mr. Windham's mind was of capacity adequate to receive instruction, and of power sufficient to act on such instruction for the government of himself and his affairs. A double question seemed thus to arise. Without burdening our pages with hackneyed quotations, we venture to propound, as a psychological axiom, that to constitute a sound mind, perception and capacity of action according to that perception must be identified. The petitioners adduced facts to negative either assumption. They were met by the doctrine of Lord Hardwicke, though the case was not quoted: "There may be a weakness of mind that may render a man incapable of governing himself from violence of passion, and from vice and extravagance, and yet not sufficient, under the rule of the law and the constitution of this country, to direct a commission."* Without detracting from the wisdom of that most learned judge, we refer to the criticism on his opinion which Lord Eldon supplied in the case of Ridgway *v.* Darwin,† to which we purpose to direct attention. The adoption of Lord Hardwicke's dictum by Mr. Windham's counsel, as a principle of law rather than a matter of opinion, begged the questions which the issues involved. Sir Hugh Cairns, in an address as brilliant as it was exceptional, urged those maxims of English law which, to the initiated, were but platitudes expressive of the right of free-born men to free action, and relied on quotations from Blackstone, with other authorities, in support of the proposition, that prodigality which causes great houses to fall and hereditary estates to pass away is "not a little conducive towards keeping our mixed constitution in its due health and vigour." Lord Hardwicke's doctrine and passages quoted may be received as an

---

* *Ex parte* Barnsley, 3 Atk., p. 172.
† Ridgway *v.* Darwin, 8 Vesey, p. 65.

exposition of the judicial spirit at a time when mental diseases were but little understood. On the part of the petitioners, the expressions of Lord Eldon were advanced. These expressions were uttered when the court had greater experience in such matters. "I have reason," says Lord Eldon, "to believe the court did not, in Lord Hardwicke's time, grant a commission of lunacy in cases in which it has since granted. Of late, the question has not been whether the party is absolutely insane, but the court has thought itself authorized (though certainly many difficult and delicate cases with regard to the liberty of the subject occur upon that) to issue the commission, provided it is made out that the party is unable to act with any proper and provident management, liable to be robbed by any one, under the imbecility of mind *not strictly insanity*, but as to the mischief calling for as much protection as actual insanity." The petitioners did not seek to place their case higher than this. They contended that it came within the category of the instances to which Lord Eldon referred, and which Lord Hardwicke foreshadowed in the judgment quoted, when, regarding the then comparatively limited intimacy with the anomalies of mental weakness, and the unyielding nature of English legislation, he further observed:—"Possibly the law may be too strict; and it might be useful in some cases, that a curator or tutor should be set over prodigal or weak persons as in civil law."* This, and this only, the petitioners asserted, was the object they sought for in the Commission, and in seeking this step they had the additional authority of Lord Thurlow, whose expressions in Living *v.* Calverly† inclined to the establishing of a *curatio* over a person incapable of managing his affairs.

On the questions of facts pertinent to the issue, it would be straw-splitting to say that on either side any essential difference existed. On the signification of the facts the inquiry hung. There was a choice of an alternative. Either alternative was a sad one! On the one hand, the petitioners contended that actions and expressions admitted, were inexplicable except on the assumption of mental weakness. On the other side, it was affirmed that not deficiency but depravity of mind, not insanity but vice, solved the problem. The petitioners included the nearest and dearest members of the family, whose interests and character were involved. There was but one exception. Mr. Windham's mother, as her counsel, Mr. Charles Russell, ably and eloquently expressed it, "gave all her sympathies, interest, and wishes to her son." Of this lady we shall only

---

* 3 Atk., p. 172.

† *Pr. Ch.*, 229. *Gil. Rep.* 4.

remark, that to our mind the impression which a review of the whole case affords, and our inferences from the affidavits which have appeared, render it a cause for regret that her conduct in the matter admitted of even an imputation. We have no wish to press an inquiry, or too closely investigate charges painful to all, and, as we believe, especially to her, who, if she erred in her judgment, we trust has done so through a mother's love and woman's weakness. Whether she still has confidence in the future of her son we know not. Time will soon declare the truth. The case is not, and was not, one for harsh expressions. It is true that the affidavits sworn in its progress exhibited a strange conflict of interest and principle on the part of one from whom different proceedings might have been expected. But where all was exceptional and anomalous, it is better for the purposes of present discussion to accept as a whole the body of evidence, and to test the several parts in their relation to the whole as well as to each other. It is a narrow philosophy that expects in worldly matters other than a conflict of interests and a difference of opinion. When opinions are inferences from facts, their value ought to be determinable according to the capability of those who give them expression, to estimate the facts as well as to form the opinion. Adopting this view, we enter on a rapid survey of the history of Mr. Windham previous to his submission to medical inspection for the purposes of determining his mental condition.

Mr. Chambers, in his able opening address, stated that "from earliest infancy Mr. Windham was not like other children; he had not the usual intellectual powers; in fact, his case was one of mental deficiency—not of absolute mania, or lunacy with delusions, but of imbecility." Mr. Nichols, a surgeon of high repute, having special experience in the varied forms of mental disease, declared in evidence that he was consulted by Mr. Windham's father in reference to his (Mr. Windham's) condition, when he was a child of but four years of age, and that he then considered him to be of "infirm organization." His physical constitution was defective. He presented a conformation of palate met with in a large per-centage of imbeciles. Mr. Nichols expressed an opinion to his father then, and seventeen years afterwards he is called on to repeat it. Why? Because his prognostications have been to this extent verified, that in the estimation of many the adult child was of incompetent mind. We will not press the question beyond the judgment of the seven dissentient jurors.

As the boy progressed, it became at least evident that his mental constitution was peculiar. On this point all the witnesses agree. In what did its peculiarity consist? We venture to suggest—in a want of due relation between the moral and intellectual sense: giving rise, on the one hand, to cunning as distinguished from capacity; on the other, to grossness as something different from vice. Was this congenital, or the result of defective rearing and education? We incline to the former opinion, and are strengthened in this view by a consideration of the means adopted for his education from early youth to manhood. From four to six years Mr. Windham is described as "high spirited," requiring a great deal of chastisement at his lessons, which he did pretty well, much better in witness's opinion than some other pupils.* An only child, his father and mother treated him very indulgently. The servants petted him. "Poor little fellow, let him amuse himself; he has no playfellows,"† was the ready excuse of fond parents for every folly. He was partial to the society of servants, and imitated their habits. His father, as it is affirmed, to shame him out of the practice of waiting at table, procured him a suit of livery. It seems to have had the contrary effect, for he continued to wear it until he was unable to longer put it on, recurring to its use on his return home from the several schools, including Eton, to which he had been sent.

Even at this period there is a significant fact which pervades Mr. Windham's entire career—an absence of suitable companionship. His having no playfellows was a matter of regret to his father; and yet "there were some young children belonging to the families in the neighbourhood,"‡ but no one of them was ever seen at the Hall, nor was young Windham invited to their association. Why was this? Considering the squire's position, the reverse might reasonably have been expected. Mr. Bickmore's evidence may possibly afford a clue. It was not imputed to Miss Rauschen, the nursery governess, who had the charge of Mr. Windham prior to his school-days, that she was deficient either in judgment or capacity. She seems to have managed him well, as far as was practicable, and for more than two years to have had the principal responsibility in the rearing of the "high-spirited boy." Shortly after she left, the youth was sent to school, where he continued for two years. The number at the school was limited. The heir of Felbrigg might be justly regarded as a most desirable pupil; some of his connexions were being educated at the same time with him. Mr. Bickmore and his wife declare him to have been amenable to authority, "no trouble in managing him," and yet, long before the period at which other pupils were removed, and while the purpose for which he was sent was far from being accomplished, "when he was not sufficiently advanced for Eton," Mr. Bickmore, at the

---

* Miss Rauschen's evidence. † Martin's evidence. ‡ Ibid.

risk of offending an influential family, and at the certain loss of a pupil of consideration, refused to keep him longer at the school. Contrast what Mr. Bickmore said with what he did. The contrast struck the jury as peculiar. They further sifted the question of his dismissal. Mr. Bickmore would fain have conveyed the impression that it was for the sake of the youth's association with more advanced boys—an assertion negatived by the fact, that boys from three to four years older were at the time permitted to remain. The real cause slipped out on cross-examination: "Another reason for his wishing the boy to be removed was, that he had heard young Windham had used violent language and oaths, and he did not think it would be desirable to expose other boys fresh from home to the influence of such an example." At this time he had been for more than two years under the careful tutorage of Mr. Bickmore. He had been placed there before his eighth year with a mind fresh and impressionable. He had previously been, for a similar period, under his governess's charge. It is fair to presume that negligence was not attributable to either of them; and yet, to rest the proposition on simple fact, their best exertions failed, on Mr. Bickmore's showing, to render the child a suitable, not to say safe companion, for boys of his own age. We dwell on this period of Mr. Windham's history. Much stress has been laid on the influence of his early home education. "Old Mr. Windham," it is said, "was a very passionate man, but his passion was soon over." "His treatment of his son was very irregular and capricious; sometimes he was indulgent, and sometimes severe."* During the short period which interposed between the leaving of his governess and his being placed at school, his mother appears to have devoted much attention to his guidance. "When he was about seven or eight years of age, Lady Sophia was on a visit at her father's house at Brighton; her son was with her; she seemed very anxious respecting him. Her task was no easy one. Her means of control were significant: when she came down with him to luncheon, her constant practice was to carry a little whip, to keep him in order."† We seek not to attach more importance to these statements than that which they imply. The boy was unusually difficult to manage, and his parents felt that it was so. In consequence of this, from a very early age they sought for competent and careful persons to educate and guide him. His youth was spent removed from the over-indulgence of home. The faults of bringing-up, on which so much stress has been laid, so far as the schools went, he experienced in common with many; and, so far as home was concerned, when vacations afforded

* Martin's evidence.

† Marquis of Bristol's evidence.

opportunity, were few and far between. His management at that time can only, therefore, be quoted as showing the estimate of his character and capacity by those best acquainted with both. In our censure on the living let us not be unjust to the dead. The faults and peculiarities of the father are advanced as arguments in justification of the son. Would that he had followed in his father's steps! He might then, indeed, have proved "not altogether unworthy of the name which he bore, and of the social position into which he was born."

Previously to his being placed at Eton, Mr. Windham may be said to have manifested in their combinations an aggregate of the irregularities common to undisciplined youth, together with a marked fondness for low company, and an habitual disregard for truth. On these points witnesses agree. Let us not seek to give too much importance to isolated occurrences detailed, such as that which occurred during luncheon at Lord Bristol's, and yet it is an instance symptomatic of a certain mental condition. The youth wanted some tart, and, instead of using a knife or spoon, he thrust his hand into the dish and pulled away crust and fruit together; when, being remonstrated with by his mother, and told by Lady Alfred Hervey he should have none, he seized a large carving-knife and rushed at her. An unmannerly and passionate, yet sane boy, might have acted similarly. The occurrence is valuable as one of many incidents, and acquires additional importance from its association. Neither should undue weight be attached to his childish predilection for imitation of railway trains beyond this, that up to the issuing of the Commission, Mr. Windham maintained the same tastes, and gratified them in a manner we shall presently discuss. The facts admitted at this period may be briefly epitomized:—A mental development which attracted the early attention of his father, and elicited the opinion of an experienced physician; and great difficulty in management and training, with marked indications of a depraved rather than a vitiated moral sense, exhibited in the frequent use of language, which, in Mr. Bickmore's judgment, rendered him an unsuitable companion for youths.

With Eton a different era commenced. He was submitted to a supervision, association, and system of education distinct from that he had previously experienced. A difference of opinion exists between the witnesses who speak to this period. A careful analysis of their testimony, in its conflict, places Mr. Windham amongst a class of inferior boys, deficient in application, of a capacity below par, of dirty personal habits, subject to fits of excitement and passion, fond of the company of menials, exhibiting peculiarities which distinguished him from others in his crying, laughing, and general habits. To the sobriquet of

"Mad Windham" we direct attention because of the evidence it affords that his peculiarities marked him from his cotemporaries. Much stress was laid upon the fact that before leaving Eton he had passed up to the fifth form, accessible only to those capable of mastering certain details, which, it was alleged, were inconsistent with the theory of the petition. This resolved the necessity for the Commission into a question of experience and opinion. Thence arises the inquiry, is the capability of mastering details whereby the intelligence is abstractly employed, any safe guide to the capacity for conduct which involves the practice of discretion, and demands the exercise of the moral as well as intellectual functions? On this point medical theorists are at variance. The variance is attributable to the conflicting views which psychologists entertain. Those who hold the mind to be one and indivisible aver that the capacity tested in one direction involves a reciprocal capability in another. Those who advocate a different view believe that of necessity no ratio can be assigned to the relation between the intellectual and moral faculties, and that the perception of differences does not imply a power of their adoption. When doctors differ, to what tribunal is it best to appeal? We suggest practical experience of life, tested by the rule of common sense. We can find no expression of opinion more applicable to Mr. Windham's then condition, than those which fell from Sir John Nicholl in the case of Ingram *v.* Wyatt,* when discussing imbecility of mind. "In order," observes that distinguished judge, "to arrive at the true meaning of imbecility of mind, we may resort to what the law calls perfect capacity, which is most correctly found in the course of our pleadings. The averment to be contained in a common count is, that the testator was of sound mind, memory, and understanding, talked and discoursed rationally and sensibly, and was fully capable of any rational act requiring thought, judgment, and reflection." Here is the legal standard. "When all this can be predicated of the person, bare execution is sufficient; but if it cannot be truly predicated, a deficiency of capacity exists—a deficiency not necessarily rendering the person intestable, but in proportion to the degree of deficiency requiring clearer and more direct proof of the unbiassed testamentary intention. *Imbecility and weakness of mind may exist in different degrees between the limits of absolute idiocy on the one hand, and of perfect capacity on the other.*" Few men could speak with greater authority than Sir John Nicholl. He further observes:—"When imbecility is original, or, as medical authorities express it, connate, the memory is often perfect, especially of trifling and simple circumstances, though the

---

* 1 Hagg, *E. R.* p. 401.

other mental powers remain infantine; or, as the same authorities suppose and express it, 'the brain has never developed itself.' In such an individual the understanding has made little progress with years, it has not matured and ripened in the usual manner; yet even in such individuals, unless the imbecility be extreme, some improvement will have taken place, some progress or knowledge beyond mere infancy will have been made by the help of memory, by imitation, by habit. Such an individual will acquire many ideas, will recollect facts, and circumstances, and places, and hackneyed quotations from books, will conduct himself orderly and mannerly, will make a few rational remarks on familiar and trite subjects, may retain self-dominion and spend his own little income in providing for his wants as a boy spends his pocket-money, and yet may labour under great infirmity of mind, and be very liable to fraud and imposition." Truth is perpetually true. These observations, applicable to the Windham of that day, are pertinent to the Windham of this, because they are in accordance to actual fact and observation, however some medical men of small experience and no authority be found to question them. Let us, bearing these quotations in mind, apply the standard we have proposed to the facts adduced in evidence. At Eton, Mr. Windham was subject to careful superiors. He had all the influence of special tuition and suitable association. They failed to impress him in a manner similar to others. The pastimes of his youth assumed a more determined form. His mimic trains were exchanged for real ones. Railway-guards and porters were, when opportunity afforded, his intimates and companions. To drive a railway-carriage appeared to be the height of his ambition, and to this his mind seemed attracted with an earnestness inexplicable on the assumption of complete self-possession. It was contended on his behalf that this taste was not indicative of a low form of childish capacity, but rather a manifestation of a similar motive to that which induced Lord Claude Hamilton and other noblemen to attend lectures, and in a spirit of scientific inquiry investigate the principles directing the operations of steam machinery. It was even urged that "it requires a good deal of skill, self-possession, coolness of head, and delicacy of touch, to drive a railway-engine." For what purpose did Mr. Windham pursue it? He dressed as a guard; furnished himself with a whistle which he blew on all occasions; "sometimes sorted the parcels in the carriage, and did the work remarkably well;"* sometimes treated the drivers at the stations; and on occasions, when working on the van, in company with the

* Ford's evidence.

genuine official and his friend, indulged in champagne to the extent of a bottle each. At the stations, running up and down the platforms, opening the doors, attending to the passengers, crying out the names of the different places, and so deporting himself that, in the opinion of more than one railway-inspector, the conclusion arrived at was, "he was short in his intellect."* This is not the method of investigation usually followed by scientific inquirers. It is worthy of notice that as Mr. Windham's capability of acting independently became established he more freely indulged in this and similar practices, which, however explicable on the part of a school-boy, is, to our mind, when pursued by an adult under such circumstances, demonstrative of no other mental condition than that set forth in the case of Ridgway *v.* Darwin already detailed, and in the opinion of Sir J. Nicholl just quoted. We direct attention to the fact that neither in the management of the engine, or any single act which required either sagacity or experience, was Mr. Windham ever permitted to take any part. On one occasion only he attempted the initiative in starting a train. It was then by blowing his whistle as guard, not managing the machine as driver. "It was the day of the Bury Cattle Fair, and I was directing horse-boxes and cattle-vans to be attached to the rear of the trains. While so engaged I heard a whistle, and on looking up saw a train in motion. The same moment I noticed Mr. Windham coming up the platform, wearing a railway-guard's great-coat and belt, and carrying a whistle in his hand. He was in the act of putting the whistle to his mouth a second time when I struck it away. Had he succeeded in blowing the whistle a second time the train in motion would have stopped, and it would have been run into by an approaching train. When the guard came up I asked him where his belt was? Without answering me he said to Mr. Windham, 'I told you not to take my belt. Mr. Goody will report me for letting you have it on.' I told Mr. Windham he had no right to be about the line, and asked him to get into a carriage. He replied, 'Oh, you be ——; I am your master.'"† This evidence is somewhat in advance of the narrative, as it applies to a later period than school-days.

With Mr. Windham's departure from Eton the most important period of his life commenced—that which was more immediately to prepare him for a position wherein complete freedom of action would be his. It is a period deserving of attentive consideration, for whatever differences of opinion existed on the part of Mr. Windham's Eton associates respecting his mental capability, there

---

* Ford's evidence. † Goody's evidence.

seems to have been a unanimity in regarding him as being in many respects exceptional. Time seemed to have exaggerated the peculiarities of his childhood.

On the death of his father, in his fourteenth year, he was placed under guardians. On the departure of his uncle for India, he became a ward in Chancery. Subject to the approval of Vice-Chancellor Page Wood, tutors accompanied him to Eton. The Vice-Chancellor, in the further exercise of judicial discretion, deemed it advisable that before coming of age he should travel. He accordingly left Eton in his seventeenth year. The Rev. Thomas Goodwin, in his evidence, informed the court the extent of the information of the fifth form boy at this period. "I attempted," said the reverend gentleman, "to instruct him. His education was exceedingly deficient—not such as I should have expected in a person of his years. He knew the meaning of a word here and there in Virgil, but could not construe a single sentence correctly." Virgil was one of the books, a proficiency in which, Mr. Windham's counsel alleged, was essential for the fifth form. The tone of his mind and general demeanour at this time is also more particularly described by his guardian, Lord Alfred Hervey. "At Torquay," the period spoken of by Mr. Goodwin, "when he was seventeen years of age, it was deemed prudent to vaccinate, for the second time, every person in the house. He cried like a child the whole morning, and we had the greatest difficulty in inducing him to submit to the operation. We were compelled to threaten him." "He cried like a boy of five or six years when Vice-Chancellor Wood proposed that he should be taken abroad. He had then an idea that General Windham was urging the Vice-Chancellor and myself to get him out of the way by poison or otherwise, in order that his uncle might obtain possession of his estates. I attempted to disabuse his mind, but in vain." We shall return to this impression when investigating Dr. Buck's evidence. A year later, May, 1858, Mr. Windham being in his eighteenth year, Lord Alfred Hervey further states: "About May, 1858, I remember him staying at Lord Bristol's, in St. James's-square. At dinner he behaved himself with great propriety *for him*, so much so, indeed, that he gave me hopes he was improving; but immediately he left the dining-room he disappeared, instead of joining the ladies in the drawing-room. He did the same thing for two or three days running, and we could not discover how he disposed of himself. At last I returned to the dining-room after dinner, and I there found him with my father's footman. He had his jacket off and his shirt-sleeves tucked up quite high, and was helping to clear the things away." This is but an example of similar transactions spoken to by other witnesses. In May, 1859, Colonel Bathurst was induced to accompany Mr. Windham abroad. He remained with Mr. Windham for three months. They travelled through Switzerland. Other evidence refers to this period. Colonel Bathurst says :—"I returned to England sooner than I intended, because I felt I could do no good with him. I consider him a person of weak intellect. He may go into society with safety, though he will always conduct himself as rudely and boisterously, and make himself conspicuous, as he did with me; but his want of mental power will always render him incapable of managing his own affairs." Mr. Bruce, who met Colonel Bathurst and Mr. Windham while on their tour, though believing the latter "to be a person of perfectly sound mind," declared him to be "uneducated and uncouth in his manners, and more like a groom than a gentleman, in the habit of using coarse and filthy language, and of laughing rather loudly." Mr. Whiteside, who, during a fortnight, formed one of the same party, considered him to be an imbecile. "He had been slightly acquainted with Mr. Windham at Eton. At Lucerne, Mr. Windham came to me, slapped me on the back, and asked to be allowed to travel with my party. His request, when backed by Colonel Bathurst, was acceded to. He was with us more than a fortnight. There were two ladies in the party. He had a railway whistle and used to blow it when the party started in carriages or on mules in the mountains. At meals he hooted, and laughed, and shouted, so as to frighten people. His manner of eating was most offensive. When laughing, the food would fall from his mouth, but he generally took it up and put it back into his mouth. He slavered at the mouth. I have heard him shouting and bawling when a hundred people were present. Apologies had frequently to be made for his conduct. He often swore in the presence of ladies, and on one occasion he called out to me in a public room, 'Oh, you be ——.' I had been merely trying to prevent him tripping up a waiter who was carrying plates across the room. When travelling in the mountains on mules, Mr. Windham called the attention of the ladies to the animals in an indecent way. The party tolerated him so long out of sympathy with Colonel Bathurst." Mrs. Wilkinson, who met them abroad during this period, speaks of her having "taken a great interest in him (Mr. Windham), because she considered him to be a person of weak intellect," and detailed his conduct in general society at Spa to have been of a character which necessitated her frequent explanations and excuses on the ground that he was *mal timbré*. Considering this testimony, and adopting the least unfavourable view of Mr. Windham's conduct as here shown, the inference from the corroboration of these witnesses is obvious. Association and education had alike failed to exercise any beneficial influence on the subject of the petition. He, grown to man's estate, ranked amongst unruly, unmannerly youths; was controlled by the same means, and amenable to the same influences. Mr. Bruce, who believed him sane, detailed an incident which occurred in the Black Forest. "On one occasion when he (in his 19th year) was misbehaving himself, Colonel Bathurst knocked him down, and he blubbered like a child. Mr. Windham called Colonel Bathurst 'a —— aristocratic humbug.' The Colonel told him if he repeated such language he would knock him down. Mr. Windham repeated it, and the Colonel knocked him down." This evidence was adduced in explanation of Colonel Bathurst's observation, "One day in the Black Forest he screamed for half an hour. I concluded he was letting the steam off." Of his capability of managing money matters, previous to and at this time, Lord Alfred Hervey says, speaking of an antecedent period: "His monthly allowance (5*l.*) would be all spent the first day or so, and he never could give any account of what he had done with it; he never seemed to have a sixpence in his pocket." Colonel Bathurst's evidence on this point goes to this extent: "While we were abroad, I spent for him at the rate of about 200*l.* a year. I gave him money when he asked for it. He never had anything to show for the money he spent." Colonel Bathurst resigned his appointment. Mr. Horrocks was induced to accept the responsibility. This gentleman, while corroborating the testimony of Colonel Bathurst, adds much that is important: "He (Mr. Windham) was happiest in the lowest company, and used to boast to such persons of his great property and expectations. He also told them that there was a conspiracy on foot to defraud him of his property; for that was an idea he entertained when I was with him. Upon one occasion I told him that if he did not alter his habits and conduct he would be put into a lunatic asylum, and all his property would be taken from him. He began to cry like a child, and said he would become a guard or a porter on a railway, 'and so,' he added, 'get my 18*s.* a week.' I could not control him in any other way than by threats and severity. I was compelled at last to thrash him." This evidence, while confirming the testimony of Lord Alfred Hervey respecting the impression on the youth's mind that his uncle was anxious to act unfairly towards him, is of even greater importance when considered in relation to the evidence of Dr. Buck, who speaks to the existence of the same impression at a time when he was executing deeds and transacting business involving his uncle's future as well as contingent interests. Mr. Horrocks speaks of an incident which occurred when Mr. Windham was entering his twentieth year. He desired Mr. Windham to attend lunch in the dining-room; he refused to do so; an altercation ensued. Mr. Horrocks gave evidence: "I boxed his ears; he threw himself down upon the floor, buried his face in a mat, screamed and yelled at the top of his voice; his mouth was bleeding; his face was covered with blood and dust; Mrs. Martin led him away to be washed." Mr. Windham when examined on this matter, said that "I was hit violently on the head and knocked completely over on the stone pavement; I fell on the mat; it is lucky I did; of course I screamed." Whichever version be the correct one, his face was washed, and he did join lunch, having previously blubbered like a great schoolboy, rubbed his face in the mat, and deported himself in a manner which was aptly described as childish. It was argued that Mr. Horrocks had acted harshly and injudiciously towards his pupil. The only witness questioned on the point was Mrs. Martin, who had the largest opportunity for observation. She deposed: "Mr. Horrocks used to want his pupil to read more than he was disposed; but they went out a great deal together, and, on the whole, she thought got on very well."* On Mr. Horrocks leaving, Mr. Peatfield, a gentleman of the highest standing, on the fidelity of whose evidence no imputation was even attempted, was appointed tutor and companion. He remained with him till after he became of age, and resided, during the period spoken to, at the Lewellins', in Duke-street. He says: "While lodging in Duke-street, St. James's, I was requested by his guardians to let him be his own master." This explains how it was that when apparently under direction, at this period he acted in the manner deposed to. Mr. Peatfield and Mr. Windham travelled together in Scotland and Ireland, and subsequently visited the Channel Islands. He confirms the evidence of the other witnesses. Describing his visit to Edinburgh, while lodging in a private house in Hope-street: "We stayed there for a week; his (Mr. Windham's) conduct was noisy and boisterous. Complaints were made to me by the landlady that the neighbours had been annoyed by his opening the windows and shouting and shrieking at the top of his voice. Complaints were made of his irrational conduct generally. I remonstrated with him, but in vain. He behaved in the streets in a way I can scarcely describe. He would hiss, spit, shout, scream, laugh, roar out foolish remarks, such as 'You're a duffer,' and 'I'm all there when I'm wanted,' finishing up with what he called 'chaffing the bobbies,' meaning the policemen. He was subject to fits of passion; his face would flush, and he would use the grossest language. He would break into these passions without any apparent cause, and while the fit lasted he seemed unable to control himself. On many occasions he behaved in a terrible manner, shouting, swearing, jumping about, opening windows, and yelling into the streets. He ate with great voracity. I have seen him choke from his mode of

---

* Mrs. Martin's evidence.

eating; and he has told me he has been sick." In answer to the jury this gentleman observed: "While travelling I paid the bills. He took an interest in the expenditure, and understood that the money was coming from his own estate. He always said he liked me. Mr. Windham was at this period in all essential respects his own master." He appears from the evidence to have fully availed himself of the permission. The Lewellins, at whose house in St. James's-street he was stopping, and whose testimony was severely commented on, affirmed no more than the three companions and tutors who had the responsibility of his charge. They certainly did enter with great minuteness into many particulars of a disgusting and painful character, but for the purposes of the inquiry place the evidence no higher than the testimony of others. The police deposed to his conduct during these months being of such a character that several members of the force regarded him as childish;* that the women with whom he sought to interfere, would say to him, "Go away, you fool; you are not right in your head;" and that they, the police, never took any notice of him, or did not, when committing irregularities such as they deposed to, take him into custody, because they did not consider he was right in his head.† The evidence of one of the police-inspectors is important:‡ "Two or three months ago, he (Mr. Windham) told me that his mother had married a young man, and that they and the old General wanted to rob him of his property. He was crying at the time, the tears flowing from his eyes, and a sort of froth and foam running from his mouth. He did not use a handkerchief, but wiped his face with the cuff of his coat. I told him to go home—it was in the Haymarket, about two o'clock in the morning—and I then left him." It may be well to here recal the evidence of Lord Alfred Hervey, spoken to by other witnesses in a similar tone: "General Windham had invariably treated him with great kindness." The only evidence to show that any difference at any time existed between uncle and nephew is the evidence spoken to by Mr. Lewellin, who says, "The General came to remonstrate with his nephew about his keeping company with the police. I heard the General say, 'I will not allow you to keep low company; I have given you the reins three months before your coming of age, and I trust you will behave like a gentleman.'" Mr. Peatfield confirms this witness in the fact that though he resided with, he did not seek to exercise any control over Mr. Windham during the period spoken to.

At this time an incident is detailed worthy of consideration. The Rev. T. J. Baty, on one occasion in May, saw Mr. Windham alone.

---

* Holden's evidence.

† Oliver's evidence.

‡ Holden's evidence.

"The latter came into the room with nothing on except a dressing-gown, which was very loosely thrown about him. He was in an excited state, and told witness that he was engaged in a serious business with the detectives. He said it was impossible to mention it, as it was a private matter. He then sat down and wrote a note. After he had done so, he told the witness, in a wild way, that Sergeant Joy, of the detective force, was following the steps of a lady to whom he was engaged. He said, he believed she was unchaste, and he had employed Joy to discover the truth. He mentioned the lady's name; she was a lady of position, and had since been married to an officer in the army." This occurrence is deserving of careful consideration, especially when we connect it with other testimony.

Dr. Tuke, in his examination by Mr. Karslake, when detailing his interview with Mr. Windham, stated: "On one occasion he, Mr. Windham, said, 'This marriage of mine is all my uncle's doing. He would not let me marry a young lady to whom I was much attached.' He described her as the daughter of a near neighbour of his in the country. He added, 'Things would have been very different if I had married her.'" An assertion of this character was, of all others, one most calculated to excite prejudice. We will avoid Dr. Tuke's example, "who did not test the truth of Mr. Windham's statement about his engagement to a young lady in the country," and endeavour to see of what importance it is deserving. In the first place, the assertion stands without the slightest confirmation, and in the next, the inference it suggests is at variance with the facts. The period spoken to by Mr. Baty was in the month of May. It was shown on the application to the Lords Justices in "Windham *v.* Giubilei," that in the month of June Miss Agnes Willoughby, *alias* Rogers, informed Mr. Bowen May that "Mr. Windham, a young gentleman of fortune, had made her an offer." At the date of Mr. Baty's visit, Mr. Windham was on intimate terms with that lady, and urging his suit. Mr. Windham's counsel did not hesitate to put forward the evidence of Mr. Andrews to speak of a boyish attachment of Mr. Windham's in his seventeenth or eighteenth year. Can it be doubted that, if there was the slightest foundation for such an assertion as Mr. Windham's uncle having prevented a desirable match, it would not have been paraded with all that delicate energy which characterized the other proceedings? In the absence of such evidence we are bound to regard the assertion as being incapable of proof. The value to be attached to the arguments of counsel, that he (Mr. Windham) was a youth capable of a virtuous and reputable attachment, who might, if better managed, have turned out otherwise, falls to the ground, when we call to mind the sworn and uncontradicted testimony of the witnesses examined on that particular part of the case. Mr. Cheales says: "Ladies have asked me in the presence of Mr. Windham to protect them and take them away. He was annoying them by rudeness—following them about the room, shouting, and trying to get hold of them; probably in sport." Colonel Bathurst adds: "He was always falling in love with ladies whom he met, and to some he made proposals. Complaints were made to me of his having used filthy language in the presence of ladies. I induced him to make an apology." Mr. Farrar describes his conduct at Spa: "He was particularly fond of thrusting himself upon ladies, and at the gaming-table he used to sit with his arms stretched out looking into their faces instead of attending to the play." Mr. Horrocks declares, "Whenever he was introduced to a lady he began to make love to her, and in a few weeks, or sometimes days, would make her an offer of marriage. It made no difference whether her character was good or bad." Martin, who visited him at Rimpton, adds: "He did not tell me anything about his being in love with a young lady there. He spoke rather disrespectfully of Mr. Andrews, who, he said, thought a great deal of himself;" and further observes, in reference to his visit to Mr. Windham in town, at the time to which Mr. Baty refers, "Mr. Windham called my attention to what he termed capital articles and letters in the *Times* about the 'pretty horsebreakers;' he said, 'I would rather marry a "pretty horsebreaker" than I would a lady.'" These witnesses are confirmed by others who depose to similar facts; while the evidence of Mr. Lewellin shows that when remonstrated with for his supposed intention of carrying out his desire to marry "a pretty horsebreaker" in the person of his present wife, he replied, "I never intend to marry her, nor any such ——; I have had all I want of her, and she may stay on with Jack ——." This observation Dr. Forbes Winslow refers to when speaking of his interview with Mr. Windham: "I asked him whether he, himself, had not been intimate with his wife before marriage. He said he had not. Then I asked him, 'Have you not said so to others?' He denied that he had made any remark of the kind. Afterwards I repeated the question pointedly to him, when he admitted having said that he had had all he wanted out of his wife; 'but,' he added, 'I said so by way of a blind, not wishing other parties to know what I was going to do.'" It is useless to attempt to reconcile one part of Mr. Windham's conduct with the other. Nor shall we seek to do so. From the evidence on all sides it stands sufficiently clear that neither disappointed affections nor wounded feelings had any part in conducing to his marriage with a lady sufficiently above suspicion not to require Sergeant Joy to satisfy her admirer of her moral character.

It would serve no purpose to enter with minuteness into the details of the evidence given by the several witnesses called in support of the petition. It will scarcely be contended that they were not witnesses of truth, and had not ample opportunities for forming the opinions they expressed. The justness of their conclusions the jury has questioned. The necessity for the investigation the Lords Justices have notwithstanding affirmed. How far is the one fact reconcilable with the other? Witnesses on the part of Mr. Windham, while in all important particulars confirming and bearing out the evidence of the petitioners, at the same time gave testimony which, if not directly conflicting, it was affirmed was to a great extent inconsistent with the theory on which the petitioners had framed their case. The answer to the petitioners was this :—Admitting your facts to be true, we conjoin them with other facts which take the case out of the category in which you seek to place it. You, the petitioners, affirm a congenital deficiency of mind sufficient to establish legal incapacity: we afford illustration of capacity inconsistent with your theory, and rebut the impressions your witnesses have created by examining many who, at the same time, under similar circumstances, had equal opportunity and capability for arriving at a conclusion. Accordingly a host of witnesses are called to set aside the theory advanced. It is worth while to examine how far these witnesses are consistent with each other and with the now admitted facts. Mr. Hancock proves that from the congenital construction of Mr. Windham's palate and the conformation of his mouth, the peculiarities deposed to by many on the behalf of the petitioners, were the necessary result of purely physical conditions. In answer to this opinion others are called, who, from their opportunity of being with and seeing Mr. Windham, are relied upon as competent to speak to facts:—they say, "They never noticed anything peculiar about his voice or his laugh;"* "that there was no peculiar noise while eating;" "that there was nothing in his manner or appearance different from other gentlemen." One class deposes that Mr. Windham "was not neat in his dress; that, in fact, he was generally filthy; and sometimes, owing to the slavering caused by the defect in his mouth, his face was rather dirty:"† others, speaking of the same time, state, "His hands and face were always remarkably clean, and his clothes were particularly neat and tidy, while in his linen he was as particular as a lady;" another declares, he "had seen a little slaver at the mouth, but he always used his pocket-handkerchief."‡ One affirms him to be "habitually so rude as to render his company intolerable;" another considers that "his manners were

---

* Anne Thurston's evidence. † Mrs. Bickmore's evidence.
‡ Thurston's evidence.

uniformly regular and 'gentlemanly." One declares him "to be extravagant and foolish in all matters appertaining to money;" another swears she "never saw him at all extravagant—indeed, quite the reverse; and she heard him say he had been warned by his father not to get into debt at Eton." One deemed him to have been "incapable of learning separately or making any advancement in his studies; another "considered Mr. Windham rather quick at learning than otherwise. He was not assiduous, but, if he chose, he could learn more than some others." One says, "he was boisterous and passionate," another, "he was quiet and mild." It would not be difficult to place in direct antagonism the testimony of witnesses to every period of Mr. Windham's life. In all inquiries of this character a similar conflict of opinion has been manifested. It is in the nature of the case that it should be so. We can find no better criticism on the diversity of views on the present occasion advanced than the observation of Lord Brougham, in a case which his judgment has rendered famous. The validity of a testament was in question. Substituting the term commission for will—*mutatis mutandis*—we apply them to the present case:—

"When," says Lord Brougham, "we take all the witnesses together who are called to support the case of the respondent, surely there can be no inference drawn from their testimony sufficient to answer the case on the other side. They had inferior opportunities of observation: they differ materially among themselves; they sometimes prove too much, and give evidence inconsistent with the admitted facts in the case; sometimes they give very material evidence in favour of the commission; and, finally, where they are consistent with each other and with themselves, the only result of their testimony is, that what others saw and heard, and positively swore to, they did not happen to see and hear, and so cannot swear to, but of course cannot negative; for, be it observed, there is not one single contradiction; there is no witness against the commission who contradicts the things sworn to in its support at the time and place when they are so stated by the witnesses in favour of the commission. Nothing can be more clear than that, as far as the parol evidence goes, the case for the petitioners is unanswered; nothing more clear than that the balance of the testimony is wholly in favour of the commission; nay, that we can hardly weigh the testimony on the two sides against each other, inasmuch as all that the respondent's witnesses swear may be true, while the depositions of the petitioner's witnesses are entirely credited."* The answer to this, on the part of the respondents, is obvious—"You tried the question and the jury

---

* Waring *v.* Waring. Notes of Cases, 6, p. 403.

found it otherwise." They rejected the doctrine that inability to manage affairs was consistent with such conduct, correspondence, and answers, as were proved and exhibited on the part of Mr. Windham. The law constituted them the arbiters of the question. This is true. We seek not now to question the propriety of their verdict. Our desire is to argue on facts, not conclusions; and the first great fact to be determined is,—Does such a class exist as Lord Eldon alluded to in Ridgway *v.* Darwin, when he observed, in determining the propriety of a commission, "I am pretty confident Lord Hardwicke would not have gone so far; but finding, when I came here, a course of cases establishing this authority, and feeling a strong inclination to maintain it, or that the Legislature should take means to preserve persons in a *state of imbecility,* laying them as open to mischief as *insanity?*" Is there a state of imbecility of this special character, and was there reason to believe that Mr. Windham was a fitting person to be considered within it? This inquiry involves not only matters of fact, but also questions of opinion. It is more advisable to, at present, inquire how far the opinions of reliable authorities as to certain attributes characteristic of an ascertained state agree with those attributes proved to have been presented by Mr. Windham. The facts as a whole we will discuss when considering the finding of the jury. We approach the inquiry without prejudice. Dr. Forbes Winslow, when questioned by the Master, was asked to describe his idea of the legal term "unsoundness of mind." In doing so, he aptly repeated the words uttered by Lord Eldon in the case of the Earl of Portsmouth, viz.: "A condition or state of intellect between actual lunacy and idiotcy, or such a degree of mental deficiency as would incapacitate a person for the management of himself and his affairs;"—such a condition as Dr. Conolly sets forth, when remonstrating with the Lord Chief Baron touching the case Nottidge *v.* Ripley. "There are," writes Dr. Conolly, "numerous young persons whose moral character appears for a time so perverted that their education is wholly interrupted, and they cannot be at large with safety to their own character; young men, whose entire idleness, whose grossness of habits, immoderate love of drink, disregard of honesty, or general irregularity of conduct, bring disgrace and wretchedness on their relatives; and whose unsound state of mind, unless met by prompt and proper treatment, precedes the utter subversion of reason."* Dr. Tuke says, "I told Mr. Windham himself, that I thought in four or five years he would be a lunatic or kill himself, if he persisted in such habits as those which were attributed to him in the affidavits for the petition." "If," continues Dr. Conolly, "the liberty of

---

* *Remonstrance with the Lord Chief Baron, touching the Case of Nottidge* v. *Ripley*, by J. Conolly, M.D. Churchill: 1859.

an insane person is inconsistent with the safety of his property or the property of others, or with his preservation from disgraceful scenes and exposures, or with the tranquillity of his family, or his neighbours, or society; if his sensuality, his disregard of cleanliness and decency, make him offensive in private and public, dishonouring and injuring his children and his name; if his excessive eccentricity or extreme feebleness of mind subject him to continual imposition, and to ridicule, abuse, and persecution in the streets, and to frequent accidents at home and abroad; his protection and that of society demands that he should be kept in a quiet and secluded residence, guarded by watchful attendants, and not exposed to the public." Further he proceeds—"There are men of rank of minds so ill-regulated and unsound that, if uncontrolled, they would be ever the associates of the lowest profligates, and frequent the vilest abodes of vice without shame; and women of wealth and station who will drink to excess and expose themselves to every possible degradation. No one who is so unfortunate as to have in his own family, or among his acquaintance, any person manifesting any of the forms of perverted character above spoken of, will for a moment withhold assent from the opinion that all such persons require to be treated as insane. The truth is, they are already of unsound mind, and without timely treatment and control they will become ungovernably mad, and remain so for life. I trust no parent and no medical practitioner will be deterred from the only wise course to be pursued in such cases, by the authority of your lordship or by a dread of the newspapers." We pause for a moment to inquire, "Is there a class," not strictly insane, which when subjected to a commission gives rise to "difficult and delicate cases with regard to the liberty of the subject"? Is there a condition "not strictly insanity, still laying the person suffering from it open to as much mischief as actual insanity"? Is there a form of perverted character "requiring to be treated as insane, who though not insane are already of unsound mind"? And, lastly, is there a condition in which the only wise course to be pursued is one calculated to be in antagonism to the jurisdiction of the bench, "which wise course men ought not to be deterred from by a dread of the press"? Great legal and medical authorities have thus spoken. Where are the illustrations to be found? It is true that passion may be indulged in to the most flagrant excess without transgressing the boundary which legislation has declared. It is true that society may be outraged to the last degree without the delinquent suffering more than the moral penalty which the forfeiture of its good opinion entails. The passages quoted indicate more than either of these extremes, or they mean nothing. They point to some medium state entailing on the individual the inconvenience of both conditions, and yet not involving him in the responsibilities of either. How much of vice and how little of sanity must be present to constitute this condition? Is what a man says to be tested by what he does? and if so, which shall preponderate in our judgment,—thought or action? As we may have mania without delusion, so we may have unsoundness of mind without insanity, or rather "a condition not strictly insanity." What, in such a complicity of doctrines and antagonism of influences, is to determine our opinion? Again, before we pass onward to the more extended examination of the last stage of the case under analysis, we supply an answer from a learned judge, whose authority we have already quoted: to our mind it is the only one which meets the difficulty. Sir John Nicholl, when giving judgment in an abstruse case, observed, "It is a great but not an uncommon error to suppose that because a person can understand a question put to him, and can give a rational answer to such question, he is of perfect sound mind, and is capable of making a will for any purpose whatever, whereas the rule of law —and it is the rule of common sense—is far otherwise: the competency of the mind must be judged of by the nature of the act to be done, *and from a consideration of all the circumstances of the case.*"*

On the 9th of August, 1861, William Frederick Windham came of age. He then succeeded to the immediate and absolute possession of Felbrigg Hall, Norfolk, with park, grounds, and timber annexed thereto, having a rental of 3100*l.* a-year, subject to his mother's jointure for life of 1500*l.* a-year, with outgoing expenses of about 350*l.* annually. In 1869, he would become further entitled to a nominal income of 9000*l.* in addition, which, owing to incumbrances, would not yield a positive yearly receipt of more than 5000*l.* The latter income, according to the settlements, was to be an income for his own life. He thus at his majority had 1200*l.* a-year available for immediate expenses. A few days before this important event, he determined on entering on what the greater number of men consider as the principal step in life—marriage. A few days afterwards, in the presence of the person he selected as his future wife, under the guidance of Mr. Bowen May, "a solicitor of twenty years' standing," he gives directions for his settlement on marriage to be prepared. On the 30th of August the settlements having been prepared were executed, and he was married. No time was lost by his relatives in instituting proceedings to ascertain whether this act, the most important, though not the most strange event of a three weeks' freedom from control, was the reasonable exercise of an independent judgment, contracted and entered into with a full sense of its responsibility. It became, therefore, requisite to inquire into all

* Marsh *v.* Tyrrell *v.* Harding. 2 Hagg. p. 122.

the circumstances. The public were first informed of what was in contemplation on the occasion of an application to Vice-Chancellor Wood to commit Mr. May for "an unauthorized and improper dealing with an infant while a ward of Court." As the case stood, the Vice-Chancellor declared it "to be one of grave suspicion against Mr. May," but was unable to act, because, "in this case, unfortunately, it was after the young man had been released from the protection of the Court that this unhappy marriage was contracted, and settlements of a most extravagant and extraordinary character were executed." The Vice-Chancellor's opinion of the propriety of the steps then being taken, may be inferred from his following observations: "The application was perfectly justifiable, and one which the guardian of the infant was bound, in the discharge of his duty, to bring before the Court."

It is well to review the principal incidents which induced the Vice-Chancellor to so express himself.

Miss Willoughby, we give her all the advantage of the good taste she displayed in her selection of that name instead of "Rogers," was, to adopt the words of her learned counsel, "not yet twenty-two years of age; she was a very pretty and attractive person; she had ladylike manners, and was a celebrity in certain circles in London; she had an allowance of 2000*l.* a-year, and occupied a high position in her own circle." Mr. Bowen May added, "She rode to hounds remarkably well." "The truth was," further observed her counsel, "she made a considerable sacrifice in marrying Mr. Windham; her affection for Mr. Windham was not strong." The only ground on which the assertion of this income on the part of Miss Willoughby rests, is her own statement; the only proof in its corroboration, as showing her resources before marriage, is found in the payment within a month afterwards of two sums to Mr. Attenborough of 135*l.* 4*s.* on the 9th of October, and 226*l.* 8*s.* on the 31st of the same month, "to redeem old jewelry pledged by Mrs. Windham before her marriage, for which Mr. Windham was threatened with an action."* The condition of her affections as regards Mr. Windham is best described by adopting the phrase of Vice-Chancellor Wood, as being "an actual repugnancy." He was solicitous and liberal in wooing. Her woman's nature prevailed, and she determined on "the sacrifice." The victim had a weakness; she required proofs of affection, and received them by gifts of from four to five thousand pounds' worth of jewellery, purchased at Emmanuel's between the 13th and 25th of August. Miss Willoughby was known at the establishment; "she had previously visited it with another gentleman who purchased jewellery for

---

* Evidence of Mr. Bowen May.

her." Mr. Windham proved if he was hard to love, he was not, in Miss Willoughby's hands, difficult to manage. On the 26th of August, in company with Mr. Windham, she visited Mr. Bowen May. The terms of settlement were arranged; an immediate income of 800*l.* and a prospective income of 1500*l.* a-year was settled on the intended bride; she observing, "Mind, I am to have the allowance so that I can will it away;" ridiculing the proposal of a lesser sum by the remark, "Do you think it is likely I will give up 2000*l.* a-year for such a settlement?" Mr. Bowen May, if he did not do all that might have been expected, did this: he saw Mr. Windham alone, and said to him, "Are you really in earnest about being married, because I think it is right to tell you that the lady is a kept mistress, and she is very extravagant; she keeps two or three horses, and is used to hunting." Mr. Windham replied, "I know all that, and I am much obliged to you." Where was the zeal Mr. Windham had previously manifested for the services of Detective Joy? At this period Dr. Illingworth declares that Mr. Windham was suffering from a loathsome disease, and knew, or at least had been told, that he "was not in a fit state to marry." Mr. Johnstone corroborated the testimony of Dr. Illingworth, and mentioned that he did not specially caution Mr. Windham, for, from his condition, he never contemplated the possibility of any sane man so affected, contracting marriage. Miss Willoughby called on Mr. Johnstone a few days before the event, and Mr. Johnstone declares, that "though his conversation with her was professional, it had no reference to that person herself." The day was fixed for the marriage. The evening previously Mr. Windham visited his intended bride and found her in the society of the gentleman under whose protection she was. This gentleman slept in the same house with her. Mr. Windham, who saw his boots at his bedroom door, does not believe, that on the eve of their separation, his intimacy extended further. On the morning of the ceremony Dr. Whidborne was sent for. This practitioner, the medical attendant of the bride, who had consented to be a trustee to the settlement on the solicitation of Mr. May, received a message that Mr. Windham wanted to consult him professionally. The doctor was suffering from dislocation of the ribs, and went to St. John's Wood much against his will. Mrs. Windham met him on his arrival, and said, "I am glad you are come, doctor, for Mr. Windham wants to see you." Mr. Windham informed Dr. Whidborne he "had chafed himself with a pair of tight trowsers," and witness told him it was a mere trifle, and prescribed an application of cold cream. Mr. Windham did not tell him that he had consulted Dr. Illingworth and Mr. Johnstone. Dr. Whidborne was then introduced to the wedding party, of which he does not appear to have had any previous notice, his visit having been demanded for "professional purposes." He says, "There were several men and women in the room, but the only person he knew was Mr. Bowen May. He understood that Mrs. Windham's mother was present, but he did not speak to her. He was introduced to her brother, who was dressed respectably, though rather common in his manners. There were several other men in the room, but he had never seen them before, and did not speak to them; they were between thirty and forty years of age, and were dressed well enough, but he did not think they were gentlemen." Dr. Whidborne acted as father of the bride and gave her away. THE SACRIFICE WAS COMPLETED. "Such a marriage," observed Sir Hugh Cairns, "was, no doubt, much to be regretted; it was properly distasteful to society; at the same time nobody had suggested or would suggest that such a marriage was in the slightest degree evidence of insanity, and the medical witness had said exactly the reverse." "Even in contracting his marriage," urges Mr. Karslake, "Mr. Windham had done an act which was calculated to disgust society." "The marriage," indignantly exclaims Mr. Coleridge, "from the beginning to the end, had been treated as the crowning act of his, Mr. Windham's, insanity, and in consequence no imputation had been too gross, no sneer had been too bitter, no story had been too filthy, for Mr. Chambers to bring forward in the hopes of aspersing and crushing Mrs. Windham, and with Mrs. Windham her husband." "Mr. Windham was neither the first nor the last, neither the youngest nor the oldest, neither the wisest nor the most foolish person who had been fascinated by Agnes Willoughby. If all the gossip he heard was true, his client was a woman whom to captivate and make his wife might naturally enough be an object of ambition to a person brought up like Mr. Windham." Which of these opinions are we to adopt? Sir Hugh Cairns argues from the medical testimony that this marriage was exactly the reverse to an evidence of insanity. Certainly Dr. Seymour affirmed that "inasmuch as the same action had been committed by the wisest, noblest, and richest of the land, he should not take it as a test of insanity." With profound respect for Dr. Seymour, we venture to affirm that the marriage in question, in its immediate antecedents and consequences, is wholly without a parallel. Unfortunately instances are not wanting in which men of rank and intellect have committed themselves to questionable alliances. In such cases association had led to esteem and grave errors been merged in strong attachments. We have never heard those marriages quoted as arguments in favour of either propriety or wisdom. Nor can we say there is any illustration of a man sober or sane wedding a woman who had a repugnance to him, she being fresh from the embraces of her paramour and he infected with a loathsome disease. The act of marriage was justly described by Mr. Karslake as one calculated to disgust society. Yet such an act was, from Mr. Coleridge's argument, a natural object of ambition, and therefore *pro tanto* an evidence of sanity on the part of Mr. Windham. But it is argued the case must not be made worse than it really was. Intimacy was not immediately antecedent, and even if it was, it is a sentimentalism to regard that isolated fact as indicating more than an extension of the principle that admitted the marriage. Mr. Windham's having been at the time in an unfit state, counsel contended, was a matter of accident, not only not within his knowledge, but his state was one on which his mind had been satisfied by competent medical authority. His union at the time was a mistake. What then? The mistake was another's, not his. Dr. Tuke declared "Mr. Windham told him that immediately before his marriage Agnes said, 'William, you are ill. I have been to Mr. Johnson, and I know you are.' Mr. Windham said he was not. She then asked him to allow Dr. Whidborne to examine him, and that gentleman said, shaking him by the hand, and with almost tears in his eyes, 'My dear boy, you are all right: there is nothing remaining now but a mere abrasure; you need be under no alarm.'" Contrast this statement with Dr. Whidborne's evidence, and in every important particular it is devoid of truth. Dr. Whidborne was not informed of pre-existing disease, or of other medical men having been previously consulted, while as to the cause of any appearance present he was intentionally misled. Mrs. Windham was, however, a purchaser with notice. She was in possession of Mr. Johnson's opinion. Whether she informed her confidential physician of all she knew is quite another question. She does not appear to have done so.

After his marriage Mr. Windham proceeded to Paris. There he remained for about a fortnight. On his return, according to his statement in Dr. Seymour's evidence, "he could not afford to take a house, and therefore his wife advised him to take apartments at Mr. Roberts's, in Piccadilly, Mr. Roberts being one of the trustees to her settlement. Mr. Roberts played an important part in Mr. Windham's post-nuptial career. His exact relations with Mr. Roberts, and Mr. Roberts's estimate of him, may be inferred from the following testimony: "He told me that on one occasion he had a quarrel with Roberts about money matters, and that Roberts in a fit of anger had declared that he had slept with Mrs. Windham previously to her marriage. I expressed my surprise that under those circumstances he should continue to reside with Roberts. The reply he made was, Roberts is a very good fellow, and I can see no reason why I should not consider him as my friend."* With this knowledge, or after this information, the testimony of Ford is deserving of notice : " One day in September, Mr. and Mrs. Windham and a strange gentleman went down with me in the night mail train. All three got into the same carriage at London. They were alone. At Broxbourne he asked me to lock Roberts and Mrs. Windham in the carriage, and as I was passing along the platform Roberts himself looked out of the window and requested me to lock the door. I did so. I observed that Roberts and the lady had a large dressing case, which they put in the middle of the carriage, and covered with cushions so as to form a bed. Mrs. Windham drew down the blinds of the window. At nearly every station Mr. Windham saw the state of the carriage, but never interfered."† Mr. Roberts has written a letter in his own vindication respecting this matter, in which he observes, "The evidence goes to show, which was a fact, that the state in which Mrs. Windham was when I unfortunately accompanied her in a railway carriage with a locked door, does away with any motive on my part, could I have been base enough to follow the course inferred."‡ Mr. Windham's condition during the visit of Roberts, on the occasion referred to, is thus spoken of by Dr. Gwyn, a witness relied on in Mr. Windham's behalf: "Early in September," says Dr. Gwyn, " I saw Mr. Windham in Norwich, and upon his invitation I went to Felbrigg-hall on the 23rd. Mrs. Windham and Mr. Roberts were there. Mr. Windham consulted me professionally about his throat. On the 23rd of September, I know she was perfectly well, because from what passed between us I examined her, but could detect no active disease. Since the 8th of October she has suffered very severely." While Dr. Whidborne, who visited Felbrigg-hall in company with Mr. Bowen May, and was professionally consulted by Mrs. Windham, speaks of the lady as suffering from derangement of the stomach, the result of travelling. The positive assertion of the one medical attendant, and the significant silence of the other, are strangely at variance with the reasons which Mr. Roberts so modestly puts forward in his vindication. The explanation of Mrs. Windham's healthy state as contrasted with her husband's, rested in causes explained by her physician. How and from whom was Mr. Roberts's information derived ? During Dr. Whidborne's visit Mr. Windham was taken suddenly ill and went to bed. Dr. Gwyn details his symptoms as specific. On the 26th of September Mrs. Windham left Felbrigg by her doctor's advice and with her husband's consent, no place to visit being mentioned. A few days afterwards she informed her husband by a letter from Dublin of her being in

* Dr. Winslow's evidence.

† Ford's evidence.

‡ Letter to *Times*, Jan. 21.

that city, where an operatic company, including a gentleman spoken of as her dear, dear ——, was then engaged. On her return from Dublin on the 8th of October, Mrs. Windham consulted Dr. Gwyn, who then for the first time found her to be suffering from the affection spoken of. She lost not a moment in having her feelings appeased. On the 11th of October, three days after her return, Mr. Dore visits Felbrigg, armed with 12,000*l.* worth of jewels and blank acceptances.* Mrs. Windham is appeased by the gift of 5419*l.* 16s. 1d. worth of jewels. On the 13th of October, when the doctor calls to pay his visit, he finds her gone. Dr. Seymour regards these gifts as evidences that Mr. Windham "was so desperately in love with his wife that he gave her the jewels in order to secure her affections." Counsel argued that in the selection evidences of undoubted sanity were manifested, inasmuch as the money was spent, not on "mere workmanship, but precious stones, which were always valuable." Let Mr. Windham have the benefit of both suggestions.

The date of Mr. Roberts's visit is fixed by the evidence of Dr. Buck, who attended Mr. Windham at Felbrigg-hall on the 21st of September, and "there found Mr. and Mrs. Windham, Mr. Roberts, and two sisters of Mrs. Windham, at breakfast." Let the question of date and all that it involves pass, this visit suggests more important considerations. Dr. Tuke in his cross-examination observed: "I did not question him, Mr. Windham, as to another statement in the affidavit of Dr. Buck—viz., that when Dr. Buck was sent for to see Mr. Windham at Felbrigg, in September, he was asked by Mrs. Windham whether he did not think her husband was being poisoned by his relatives?" Did Mrs. Windham think so? And if so, did she fail to impart her belief to her husband? Was there any object to gain in impressing his mind with such a conviction, and what was her estimate of a mind that could be so impressed? On the 2nd of September Mr. Windham had been induced to execute a disentailing deed of Felbrigg. Other deeds were required. His present property, with his mother's life charge, wife's jointure, and progressive debts, was well under control, his future property was worth something. Accordingly a will was executed at this time, "To the wife for life, with remainder to children; remainder again to his mother for life; remainder over to his sister-in-law; the one who married first with consent of guardians to take the name and arms of Windham."

Many witnesses depose to the existence of impressions on the mind of Mr. Windham, to our judgment of the first significance. Lord Alfred Hervey stated: "When it was proposed that he, Mr. Windham, should go abroad, he had an idea that General

---

* Mr. Dore's evidence.

Windham was urging the Vice-Chancellor and myself to get him out of the way by poison or otherwise, in order that his uncle might obtain possession of his estates. I attempted to disabuse his mind, but in vain." Mr. Horrocks adds: "He was happiest in the lowest company, and used to boast to such persons of his great property and expectations. He also told them that there was a conspiracy on foot to defraud him of his property; for that was an idea he entertained when I was with him." Inspector Holden says: "Two or three months ago (before the trial) he told me that his mother had married a young man, and that they and the old General wanted to rob him of his property. He was crying at the time, the tears flowing from his eyes, and a sort of froth or foam running from his mouth. He was crying like a child, and said everybody was trying to rob him." All the witnesses agree that Mr. Windham was proud of his uncle, and until the few weeks previous to his coming of age there is no evidence of any kind to lead to the inference that other than the most affectionate and friendly understanding existed between them. If these witnesses be believed, and the feeling of Mr. Windham towards his uncle be rightly reported, and there is every reason to affirm that they are so, what inference suggests itself to every candid mind? In our judgment there is but one answer to such an inquiry.

Dr. Buck does not appear to have fallen into Mrs. Windham's views. On calling the next day she saw him, and in consequence of what she said he left without seeing Mr. Windham. The same afternoon he received a letter as follows:—"Felbrigg, September 26th, 1861. Dear Sir,—My wife prefers me having her medical man to attend me, and he is now here. I am sorry for this; but she will have it. Yours truly, W. F. WINDHAM." It is only necessary to add Dr. Gwyn was the medical man favoured with Mrs. Windham's confidence.

It does not appear what were the exact circumstances under which Mrs. Windham left Felbrigg on the second occasion. It was known to her husband that she had proceeded to Glasgow, where the same attraction awaited her as had induced her to visit Dublin. Accompanied by Dr. Gwyn, Mr. Windham, on the 3rd of November, followed her to Glasgow. They stayed in Glasgow forty minutes, sufficient time to enable Mr. Windham to visit the Victoria Hotel, and satisfy himself that his wife had returned to London, whither he immediately followed her, and found her staying at the Euston Hotel. Mr. Wheeler, the manager of this hotel, gave evidence of Mrs. Windham's sojourn there for three days, when, in consequence of the disorder which was thereby occasioned, he requested the party to remove. Mr. Windham had previously to this had two letters, written by his wife, returned to him from the dead letter office. They were written after her marriage to some "dear darling." Their receipt does not appear to have shaken his confidence, since in his interview with Dr. Tuke, subsequent to Mrs. Windham's visits to Dublin and Glasgow, and to their perusal, he observed, "I know what Agnes Willoughby was, but I will never believe that Agnes Windham can be false to me until it is proved." Alas ! the name had not been held in honour by himself. It had failed in preserving him from shame. What influence could he have hoped it to have exercised on her?

From this period to the opening of the Commission, Mrs. Windham's career presents no features which, in their discussion, invoke the question of her husband's competency to manage his affairs, or throw additional light on the question of his soundness of mind. With this object alone have we even introduced her name here.

The competency of Mr. Windham to manage himself and his affairs was questioned by the petitioners. They affirmed the marriage was the most serious but not the most marked indication of that mental imbecility of which the other facts deposed to were but minor manifestations. Their affirmations ran somewhat in this manner: "You, Mr. Windham, have shown your incompetency to manage yourself by consorting with the lowest company, indulging in abject debauchery of every kind, consummating all by an inexcusable and disreputable marriage: your competency to manage your affairs you have shown by reckless extravagance, by disentailing your old family estate; by, in a few weeks, contracting an immense amount of debt; and also by the execution of a strange and exceptionable will."

In proof of Mr. Windham's competency to manage himself, witnesses deposed to his having on many occasions so deported himself that his conduct attracted no special observation, while in the discharge of military duties and other obligations involving the exercise of intelligence, he proved equal to, at the least, average expectations. Some placed their opinions higher, and argued that certain correspondence read was of itself sufficient to at least afford a sufficient answer to the affirmation of imbecility. Nay, more, that they implied a competency to manage, and therefore a right to mismanage his estates. "It was," observed Sir Hugh Cairns, "impossible for a man who was said to have only a limited amount of mind, or none at all, to assume at any moment or for any purpose a greater amount of mind than he really possessed. If the mind was not there, or only there in a certain small and limited quantity, no desire on the part of the individual to show a greater amount of mind, or to assume the appearance of a greater amount of mind, could supply him with that which Nature has denied him. Hence, when a man was charged with imbecility, if it could be shown that for a considerable time and in various situations he had acted like a rational being, any acts of folly which might be alleged against him should be carefully, deliberately, and keenly investigated, because at first sight it was next to impossible that a man could at certain times assume a mind and intelligence which were wholly absent." One part of these observations begs the question they open; the other involves a sophism, and affirms to be true that which does not contradict the opinion of the petitioners. Placing the proposition on no safer ground than this:—Mr. Windham was not a congenital imbecile, because he is capable of actions involving the exercise of mind. The *elenchus* is refuted by showing that both propositions may be at once true. In support of these objections, we affirm, first, that *it is* possible for a limited amount of mind to assume a character that is no index of its capability or extent, and that to argue from exceptional indications is, in the estimate of the mind, a grave error. We are also, for our second affirmative, supported by competent authority in saying that imbeciles are capable of conduct and actions apparently inconsistent with their real mental condition. The first is a matter of experience, the second a question of degree, so far as the issue involved in Mr. Windham's case. We need not travel far for authorities in confirmation. We have quoted the opinions of Dr. Conolly as to persons "already of unsound mind who require to be treated as insane." "There are," says Dr. Tuke, "absolute idiots, and there are persons afflicted with deficiency of intellect and imbecility of mind." "Some imbeciles may be educated, and be able to be taught Latin and Greek, but such cases are very rare, and where the imbecility is congenital, little or nothing in the shape of education can be imparted." Do imbeciles in learning Latin and Greek manifest the true index of their mental power, or is it the ultimate effort of a strained intelligence which memory, from constant repetition, has been able to impress? Is such a case one of those "well-defined extremes" alluded to by the Commissioners of Lunacy in their Report of 1847, respecting idiocy, where they observe—"It comprises within its limits many intermediate forms, some of which pass into each other by insensible gradations, and are not easily distinguishable by language, although the extremes are well defined and very remote from each other"? Is the observation of Dr. Copland true, "Habit has a great influence on the proceedings of imbeciles, and gives to many of them an appearance of regularity which may be mistaken for the result of steadiness and of higher powers"? And finally, to treat a practical question in a practical way, is the observation of Dr. Winslow one in accordance with experience, that "a person like Mr. Windham may so conduct himself in even a lengthened examination as to convey the impression to your mind that he is really competent to manage his affairs, but immediately afterwards he may exhibit such weakness of intellect as to prove his utter incompetence"? Imbecility of mind is not a palpable reality the physician can weigh and fix; it is, to again quote from Dr. Winslow's examination, "essentially a question of evidence and degree." We venture to affirm, in answer to the first part of Sir Hugh Cairns' proposition, that imbeciles do very frequently, through learning or strained effort, assume the appearance of a greater amount of mind than for the practical purposes of life, the management of property, or their personal safety, it would be warrantable for those interested in their welfare to act upon. Sir Hugh Cairns' second proposition is a corollary from the first, which, if put in an absolute sense, might be refuted from the general experience of those who are familiar with mental disease; but being placed as an exceptional assertion, we meet it by declaring that the case of Mr. Windham was, in our estimate of the facts, such a one as comes within Sir Hugh Cairns' class "next to impossible," and stands on that extreme the Commissioners have alluded to. But it is argued, surely the mere facts of presenting jewellery, apportioning a settlement, contracting debts, and raising money by the sale of timber, are not *per se* such evidences as would warrant the law in depriving the owner of property of his legitimate rights? If the ruin of property or its loss be a sufficient ground for so grave a step, he who stakes his estates upon the hazard of a die, by the one act consummates all in which the proceedings complained of on the part of the other may or may not eventuate. A moment's reflection shows there is neither parity of circumstances, of motives, or opportunity between the case of the gambler who loses and the imbecile debauchee who wastes his substance. True that both accomplish the same end, but by far different means. The reason of the one tells him that society has its laws founded on a reciprocity of self-restraint, and to those he attends. The other in his folly at once exclaims, "Evil, be thou my good."

The question of the timber purchase was fully discussed on the trial, and attracted much attention. Into its merits we do not care to enter. For our inquiry, it is immaterial whether Roberts was or was not honest in negotiating its sale; whether the ornamental trees might or might not have fallen beneath the axe; whether the full or an inadequate price was contracted for: we are willing to accept the transaction as an illustration of Mr. Windham's confidence in Roberts—Roberts, to whom Mr. Windham alleged to Dr. Winslow, Davis had applied a most offensive epithet; Roberts, whose declared intimacy with his wife he stated he was conscious of; Roberts, whom he still regarded as a very good fellow," and respecting whom he observed, "I could see no reason why I should not consider him as my friend," even though it were proved that he, Roberts, and not the alleged contractors, was the real purchaser of the entire timber on the estate. We pass to those further considerations which the inquiry in its progress suggests.

We have pointed out the difference of opinion which prevailed among the witnesses: let us for a moment investigate how far counsel acted on those differences. "After he left Eton," observes Sir Hugh Cairns, "he really had nothing which could properly be called education. There was not a single tutor he had who ever attempted seriously and earnestly to give him any proper kind of instruction." "From the cradle to the age of twenty-one years," continues Mr. Coleridge, "he was utterly neglected by all his family, with a set of second-rate tutors about him, who did not understand and were incapable of dealing with their pupil." What does Sir Hugh Cairns properly call education, and who were the tutors thus spoken of? Miss Rauschen had charge of him for two years in his infancy: was she negligent in her duty? Mr. Bickmore for the same time had him at school: did he fail in that which he undertook to accomplish, and if so, how, and from what cause? Mr. Westmacott, for three years, received him as an inmate of his establishment, and evidence was given by Mr. Ingram, the French and mathematical master there, in proof of his capacity, as exemplifying the care bestowed on his tuition: was Mr. Westmacott the party in fault? The Rev. Mr. Cheales was present with him at Eton: was he a second-rate tutor? and is Eton the school where he really had nothing which could be properly called education? If so, what value is to be attached to Mr. Coleridge's contention that the fifth form was attained by examination and progress, and what avails his strictures on Mr. Cheales for insinuating that in Mr. Windham's case favour was shown? Was Mr. Younge, who succeeded Mr. Cheales, incompetent for his duty? Does the Rev. Edward Hale, the present mathematical master at Eton, who, it is true, could never get Mr. Windham to learn mathematics, deserve Sir Hugh Cairns' observation? Was the Rev. Thomas Goodwin, who could not extend his studies beyond reading two chapters of Prescott's "History of Peru," equally with the others, to blame? Was Mr. Connor deserving of the same character? Is Colonel Bathurst considered by those who have the privilege of his acquaintance as a second-rate man? Was Mr. Horrocks incompetent to instruct, though unable to control? And is Mr. Peatfield, distinguished in his college and respected by his friends, to be visited with public condemnation in consequence of Mr. Windham's career? And last, though not least, are the Eton dames—those ladies whose memories are cherished by pupils of the school—to receive their share of this denunciation? We prefer Mr. Karslake's estimate of these gentlemen, though we differ with his observations respecting them. "Mr. Windham," says Mr. Karslake, "seemed to have been allowed to do what he liked in childhood: his education, such as it was, ceased at too early an age, and he never was wisely or prudently directed." "If," he further observes, "he, Mr. Windham, were a creature who spent his time in howling and catcalling, a person who never by any chance indulged in coherent and consequential conversation, would he have been kept so long at Eton, or could he have induced *gentlemen of education, ability, and position* to live and travel with him?" The only one of these gentlemen whose management was questioned on any particular point was Mr. Horrocks, and this was in consequence of the following observation he made when Mr. Windham's conduct was extreme: "You must take care, for your uncle is coming back from India, and you will be put under restraint if you do not mend your ways." It might, under other circumstances, have been argued that this remark was the strongest evidence of the sincerity of Mr. Horrocks's convictions at the time. Mr. Windham's counsel used it for another object—to explain the impressions on Mr. Windham's mind, and his antipathy against his uncle. Its value for this purpose is lost from the fact, that long before Mr. Horrocks had charge of Mr. Windham, the impressions spoken to by Lord Alfred Hervey existed. The assertion that Mr. Windham was allowed to do what he liked in childhood, and that his education ceased too early, are met by the facts that he was two years under a governess, two years at one school, and three years at another, before his father's death in the year 1854, since which period Lord Alfred Hervey stated, "though occasionally with his mother, he was chiefly under the government of tutors." Is there nothing significant in this perpetual change of instructors? Was Mr. Bickmore singular in his estimate of his pupil? Surely, if ever there was an observation warranted and sustained by facts, it was that of Mr. Chambers in his masterly reply to these calumnies: "No young gentleman ever had more care and anxiety bestowed upon him; no young gentleman of his rank ever had more money laid out upon him. Everything had been done to train and educate him, but trial after trial had failed, and his mind remained a barren soil, incapable of cultivation."

"Mr. Windham," urges Mr. Karslake in his reply on the whole evidence, "had committed many bad actions, but he was not therefore insane; and of all people in the world General Windham and the clan Bristol ought to be the last to complain of his misconduct, which was in a great measure the result of their own neglect and ill-treatment." Is this statement warranted by the evidence? We are not advocating a cause, but arguing a fact.

General Windham's conduct we seek not to question beyond the necessity of this case. Yet happily for that distinguished officer his conduct is spoken to by one whose honour, veracity, and judgment no man in the land will question. We thank Mr. Coleridge for teaching us the phrase "cruelly treated and wickedly aspersed." We apply them to the criticisms on General Windham, and in corroboration of our view we quote the observations of Vice-Chancellor Page Wood, subsequent to the finding of the jury:—"I have no hesitation," writes the Vice-Chancellor, "in saying that in this the most difficult case which I have ever met with, of the numerous wards of Court, I have derived the greatest assistance from your solicitor while you were absent on duty in India, and from your individual attention to your nephew's interests since your return. From the circumstance of your absence in India I soon felt the necessity of adding to the guardians appointed by the will of Mr. Windham's father—viz., the young man's mother and yourself. With her consent and yours I added Lord Alfred Hervey, and Mr. Hook, who had married the young man's aunt. I can speak to the deep interest which they took in his welfare. Their trouble was incessant, as was indeed my own. His mother, also, though at times taking different views as to his travelling and the like subordinate matters, never failed to attend to the proceedings. I required every possible help I could receive in appointing tutors to take charge of him. You did not return to England till many of our difficulties had been encountered, though we all failed to solve them. I recollect your letters being read to me, in which you were most anxious, if it were found possible, that he should be sufficiently educated for a commission in the army, and every effort was made to secure this desirable object, but in vain, owing to his weakness of mind.

"When you returned to England you evinced the most lively interest in his welfare, and frequently attended personally at the discussions that occurred as to the difficulties attendant on every attempt to improve the young man's character; so did also his other guardians. I can most conscientiously say that, in every respect, you evinced the highest interest in your ward's welfare. Shortly before he came of age you consulted me as to the course we had better pursue in consequence of his having formed a connexion which has since ended so disastrously. At your request, earnestly pressed upon me, I saw the young man, and gave him my advice in writing (as I found he did not correctly report anything that passed). I advised him at once to appoint a solicitor to arrange his affairs, and to travel abroad (to avoid bad connexions). He asked me to recommend a solicitor. I declined doing this, and told him to consult his guardians. He spoke very highly of you, and I said he had better consult you. You, on his doing so, recommended Mr. Jackson. The advice I gave was entirely founded on my own judgment, and he promised that he would follow it. I do not think that any guardian has ever better discharged his duty to a ward while under my charge than you did."*

The only witnesses who speak to the neglect and ill-treatment of "the clan Bristol" are the Marquis of Bristol and Lord Alfred Hervey. The former says: "Shortly after Easter in the present year (1861), Mr. Windham called at my house in London. I invited him to dine with me. He did so twice. The last time he dined with me I gave him a general invitation to luncheon, in order that if we had anything to propose to him we might do so without the trouble of making a special appointment." The latter observes: "He was always on his better behaviour with me, for I was strict with him, and he was afraid of me." While Lord Alfred Hervey added, in answer to the jury: "General Windham has invariably treated him with great kindness." The invitations and overtures extended to him were not accepted. Mr. Coleridge supplies the reason when he suggests that he, Mr. Windham, was "rough, boisterous, and riotous in his manner, probably exceedingly ill at ease with elegant and refined ladies of his own rank of life, probably not exceedingly fond of the society of ladies of the Hervey elevation." These reasons urged in vindication of Mr. Windham's infatuation for "a celebrity in certain circles" are surely so many arguments in vindication of those relatives against whose fair name and generous intentions not one single imputation has been uttered from any reliable source. We confess, as we read the details of this inquiry, we were at a loss to find from what quarter were derived instructions for accusations without proof and insinuations without reason. Sir Hugh Cairns satisfied us upon that point when he observed: "He and his learned friends had been able to elicit from the witnesses many details which they either had forgotten or omitted to state, and had obtained considerable additions to the evidence which the witnesses gave, by addressing to them proper and fit inquiries for the purpose. To whom was it supposed they could be indebted for the information which enabled them to put those questions in cross-examination? To whose shrewdness, intelligence, and clear and accurate knowledge of what really had occurred could they be indebted, except to those of Mr. Windham himself?"

There is an old adage, "He who pleads his own cause has a fool for a client." In investigations of the character we are discussing, is it not open to grave objections for counsel to accept as instructions the unconfirmed suggestions of the individual

---

* *Times*, Jan. 31, 1862.

whose soundness of mind, veracity, and character, are the special objects of inquiry? Is it not a course to be regretted when such suggestions are accepted as justifications for questioning the honour of men, and (even, as was accomplished in the observations on Mrs. Wilkinson's imputed desire to draw Mr. Windham into a marriage with her daughter) for deeply wounding the sensibilities of women? All who are familiar with the procedure of our courts of law must know from experience that principals in a suit, even when interests are comparatively small, seldom estimate their own positions with complete fairness. Counsel invariably exercise their discretion in carrying out the instructions for examination which they receive, and by doing so frequently prevent parties committing themselves to statements unsupported by actual facts. We know that such a course has the sanction of experience, and we believe it is one which is eminently productive of good. Had it on the occasion of this inquiry been followed, much that was painful to all parties might have been spared, and much that was irrelevant omitted. In adopting the instructions so received, and attributing to General Windham mean personal motives when discharging a serious and painful duty, by instituting an inquiry which, speaking from his own knowledge, the Vice-Chancellor regarded "as merely the natural sequel to all the interest he (General Windham) had shown in the youth's welfare, and as a last attempt to save him from the natural result of his disastrous marriage," in taunting him with "skulking by the side of his counsel, and avoiding the ordeal of the witness-box," had Sir Hugh Cairns justification either in practice or in fact?

Is it a matter of practice for counsel in the conduct of a cause to take upon themselves the whole responsibility of determining what witnesses shall or shall not be examined? Is it a matter of discretion to support a case by the testimony of those whose interests are most largely involved in the issue, when every fact to which they can speak is equally within the knowledge of others? The letter of Sir William Page Wood we have quoted fairly represents what the facts really were. The correspondence between General Windham and Lady Sophia Giubilei, with General Windham's affidavit since published, sufficiently answer the unsupported affirmation of Mr. Giubilei. The minds of the jury were, from the insinuations thrown out, directed to considerations beside the question at issue. We doubt if prejudice did not mislead their judgment; and that it was to no purpose the Master quoted the opinion of Lord Cottenham:—"It was said that in the verdict they gave, the jury were influenced by a suspicion that in presenting the commission against his brother, the petitioner was actuated by interested motives; but beyond the assertion of the petitioner, there is no proof that such was the fact. Doubtless it was the duty of the jury not to permit themselves to be biassed by any such feelings, however natural. At all events, such feelings cannot operate on the mind of the Court, which, in deciding questions of this kind, must be governed solely by the consideration of what is necessary for the protection of the person and property of the party."*

It may be asked—Did not General Windham meet his nephew on the footing of sanity, and seek to transact business with him? Let the two occasions to which such a question refers be fairly examined. What do they amount to? No more than this: that the day Mr. Windham came of age, *before his capacity for the management of his affairs had been tested*, a small in-lying strip of property, purchased by Mr. Windham's family for him during his minority in order to isolate his estate, was offered to him for the sum paid for it by those who wished to protect his interests.† The other proposition was a resettlement, for an equivalent in value, of the family mansion and grounds, in order to prevent that which has since occurred—the execution of a deed whereby the property might be lost before experience had satisfied its owner of its historic worth; a preventive measure which would not have curtailed Mr. Windham in raising of supplies, but which he could not understand, or if he did, could not appreciate, but the rejection of which his counsel urged was evidence of competency to manage his affairs, or at least that those who entered on the transaction of such business with him deemed him to be so. Much stress was laid upon the transaction. It afforded scope for observation seldom equalled before within courts of justice, and never on a similar civil trial, as far as our knowledge extends, approximated.

Of the numerous witnesses examined on behalf of Mr. Windham—those who saw him, and those who heard him; those who travelled with him and did not speak, those who spoke with him and did not travel—but two witnesses had sufficient opportunity for forming anything like a judgment. Marten, the bailiff, who had known him from infancy, and though thinking him competent, yet fancied his mind "a little below the average," and Sir William Foster, who had the stewardship of the estates, "who considered him competent to manage his property with help." The medical witnesses examined on his behalf did not seek to put his case higher; but they coupled their opinions with anticipations which, as part of their opinions, we receive as a test of their value. "Mr. Windham is young for his age," observed Dr. Seymour,

---

* In the matter of A. B. 1 *Mylne and Craig*, p. 541.

† *Times*, letter of Mr. Scott, Feb. 3, 1862.

"but has mind enough to take care of himself and of his property. I think this inquiry will improve him. So very solemn a process will have a steadying effect upon him. He has reason enough to profit by the lesson." Dr. Seymour did not know that pending "the solemn process," Mr. Windham, in company with the witness Howlett, and others called to testify their belief in his soundness of mind, nightly frequented the Haymarket, and with the ladies of that locality, not celebrities, discussed the events of the day in Barnes's tap-room. Dr. Hood says, "He (Mr. Windham) acknowledged that he had led a noisy and rather fast life, and regretted many of his actions, more particularly his marriage; and he told me that he should have avoided many of his indiscretions if he had known that his family were anxious to make him out a lunatic." This observation removes the supposition that Mr. Horrocks's threat had seriously influenced his feelings towards his uncle. "I endeavoured," continued Dr. Hood, "to impress upon him the desirability of acting with caution during the course of these proceedings. He did not seem to be much impressed with what I said, but he may have been so." He was not impressed, if the evidence of his conduct in the Haymarket throughout the examination is any test, and the Christmas entertainment, of which the honest Marten did not approve, be regarded as an indication.

Other arguments than those based on scientific opinions or inferences from admitted facts were laid before the jury with all the energy of zealous advocacy, and all the persuasiveness of classic oratory. An appeal was made at once to their sympathies and their fears.

Mr. Coleridge, on behalf of Mrs. Windham, in an address of surpassing ability, urged the jury to disabuse their minds of any prejudice which disgusting and filthy stories might have raised: to allow them to influence their judgment, he contended, would be "an act of great injustice to Mr. Windham, and to the woman who, after all, was his wife, to whom he was still deeply attached, and from whom no human power, save in the case of adultery, could put him asunder;" an appeal which contrasted strongly with the evidence of Dr. Hood, from whom they had just heard: "He (Mr. Windham) told me that he and his wife were not living together; that his wife had left him for other men; and that she had spent his money in a most extravagant manner. He had been greatly disappointed, and hoped never to see her again;" and he added, "When I get out of this trouble, I shall apply for a divorce." "Everything," continued Mr. Coleridge, "prized by Mrs. Windham was at stake; her marriage, her settlement, her prospects in life, her position," [that "accredited position" in society for which we presume she had made the sacrifice spoken of;] "the welfare of those two sisters of whom we had heard so much, and upon whom Mr. Chambers, though prolific in imputation, had not ventured to cast a slur—all hung upon the verdict which the jury might give. . . . He asked the jury whether they had ever heard, in the whole course of their lives, of such remorseless cruelty as an endeavour to make out a man to be mad, to take away the property in which he was interested, to separate him from his wife, to deny him the hope of children, and all the sweetest pleasures of existence, for the sole purpose of getting rid of a distasteful marriage and an abhorred settlement?" As an argument of Mr. Windham's sanity, witnesses were called to satisfy the jury that he estimated rightly his wife's conduct and contemplated a divorce. Her counsel, ignoring the evidence on that point, adopted a supposititious case and argued it with success. While in confirmation of Dr. Hood's impressions respecting the soundness of Mr. Windham's views respecting his wife, Dr. Gwyn stated, at a period long subsequent to the time Dr. Hood referred to, "I have seen Mr. Windham from time to time when he came down to this Court. I saw him speaking to his wife last Friday. Mr. Windham and I got into her brougham, and we put him down at Morley's Hotel. She set me down in Regent-street, and she went her way and I went mine." In answer to the question of the Master, Dr. Gwyn further stated, "When they were in the brougham together they were respectful to each other, and conducted themselves calmly and quietly, as any other man and wife would do." How far the testimony of one witness bears out another, and how far Mr. Coleridge's touching appeal has been supported by facts within the knowledge of all, we care not in the pursuit of our inquiry to further investigate.

Mr. Karslake took other ground. In commenting on the medical evidence sustaining the petition, he observed, "Dr. Winslow, in his own subtle way, had thrown out a bait to the jury, in the shape of a suggestion that Mr. Windham, instead of being confined in a lunatic asylum for life, might merely be placed under surveillance for a few years. Let the jury beware of falling into the snare thus set for them. They might depend upon it that the object of the petitioner, however it might be disguised, was to immure Mr. Windham in a madhouse for life. Let General Windham once get his nephew within his clutches, and he would take care that the estates were secured to his own family, and that the personalty was in a very few years divided between himself and the other petitioners." Lest any one interested in similar investigations should be weak or ignorant enough to adopt as fact these flights of fancy, we shall briefly enter on a few considerations they suggest. More than a century has passed since the rule for guidance was laid down by Lord Macclesfield: "A lunatic, in the eye of the law, is never looked upon to be desperate, but always at least in a possibility of recovering. It is his benefit and comfort I am to take care of where no creditor complains, and not to heap up wealth for the benefit of his administrators or next of kin !"*—a dictum acted on and confirmed whenever the question has arisen. "The orders," observed Lord Loughborough, "that are made by persons charged with the custody of lunatics, are appealable to the king in council. In the series of them there is one general principle, though I do not say it is without some possible deviation, that the general object of the attention of the administration is solely and entirely the interests of the lunatic himself; and with regard to the management of the estate, solely and entirely the interests of the owner, without looking to the interests of those who, upon his death, may have eventual rights of succession; and nothing could be more dangerous or mischievous than for him to consider how it would affect the successors. The Court have always shut out of their view all considerations of eventual interests, and consider only the immediate interest of the person under their care."† "A liberal application," repeats Lord Eldon, "of the property of a lunatic is to be made to secure every comfort in his situation. I do not think it is a judicious act for the Court to lay up property for persons who may be next of kin of the lunatic after his death."‡ How far these dicta bear out Sir Hugh Cairns' views of the accumulation which he affirmed would ensue for the next of kin if a verdict was found against his client, it is not our purpose to discuss. The jury were led to believe that the finding would affix a permanent stamp on Mr. Windham's liberty. They knew nothing of *Traverse de Jure*,§ by which it could be set aside whenever guidance, teaching, or more matured years, had ripened the imperfectly developed intelligence. The jury believed they would be rendered the instrument of injustice to those who had acted and traded with Mr. Windham from the period of their finding, had their verdict been otherwise. They knew not of the recognised principle that contracts are not over-reached by inquisition except on the common-law principle of fraud.|| We quote Mr. Karslake's words: "The jury did fall into the snare set for them," and as we believe sympathy was misled by sophistry, and an apprehension, from imperfect knowledge of what might be, prevented them from the full appreciation of what was. To speak of General Windham "getting his

* Dormer's Case, 2nd Peer—Williams, 264.

† Oxenden *v.* Lord Compton, 2 Vesey, 71, a. 1.

‡ Baker, exparte, 6 Vesey, p. 7.

|| Mill *v.* Morley, 9 Vesey, 478.

nephew within his clutches" was the wildest flight of imagination, while to describe the jurisdiction of the Court of Chancery as permitting such a proceeding was a bold defiance of all that those familiar with its operations are daily acquainted with. Dr. Winslow (as if in anticipation of what many months afterwards was declared by one of the most learned and sagacious judges on the Bench) rightly set forth the principle which should guide, and which does guide, the conduct of cases like Mr. Windham's, in continuing him under tutorage and protecting his property. Even as we now write, Lord Justice Knight Bruce observes, "It was a mistaken idea entertained by many, that the finding by a jury that a person was of unsound mind necessarily involved an interference with his personal freedom. The Court placed no further restraint upon a lunatic than was necessary for his protection, and several lunatics who were under the protection of the Court were now residing, with large establishments, in their own houses."* This practical fact Mr. Chambers in vain laid before the jury when impressing the opinion of Dr. Conolly, which was but a corroboration of Dr. Winslow's proposal (" subtle bait," Mr. Karslake termed it), and asking them by their verdict to commit Mr. Windham " to the care of some good, kind, virtuous friend, who would watch over him, advise him, and gradually prepare him for being able at some time or other to manage his affairs."

It is useless to continue discussion on these matters, or to enter with greater minuteness into the particulars of this case, further than a brief allusion to the observation of Dr. Conolly in answer to the jury, to which we have referred. " I believe," says Dr. Conolly, " that if Mr. Windham had one good, kind, virtuous friend, he would get through the world very well." What a climax to a medical opinion in reference to the soundness of mind of one arrived at man's estate, who had every advantage which education and wealth could bestow.

As might have been expected, medical witnesses differed materially in their estimate of facts. All agreed that an interview could not afford sufficient opportunity or material for opinion. The evidence in this case formed an element in their judgment. Those who adopted Dr. Seymour's views found in deliberate vice, debauchery, and excess of every kind, adequate explanation for Mr. Windham's shameless indifference to personal honour, and utter disregard for the esteem and respect of his fellow creatures. We presume that so far as these features are capable of presenting themselves, Mr. Windham equalled the class alluded to in Dr.

---

* *In re* Smith, heard June 13, 1862.

Conolly's pamphlet already quoted. If so, we are curious to know in what respect he differed ?

The trial was remarkable in many respects, as much so for its length, which was without precedent, as for its facts which were without parallel. The public grew weary of its details. The public press took up the popular cry. All concerned in the investigation received unqualified censure, and this in the inverse ratio of their desert, as is the common case in popular enthusiasm. The learned Master, who conducted the elaborate investigation with the deepest sense of its responsibility and the most complete impartiality, has heard his vindication from those who occupy the highest judicial positions in the country. Nay more, he has received at their hands deserved commendation. The petitioners have in their vindication but to refer to the judgment of Lords Justice Knight Bruce and Turner, when giving the decision of the Court on the petition for costs.

"The demand for costs is based on the ground that some or all of the original petitioners were actuated by improper motives, as when they presented their petition they kept back evidence, both documentary and oral, which bore upon the question of unsoundness of mind, and which, if it had been brought forward, would have gone to prove soundness of mind. It is desirable to state that the present petition does not allege malice, bad faith, improper conduct, or improper motives upon the part of the original petitioners, nor has Mr. Windham made an affidavit to the effect; and it appears to me that the evidence before us, or before the jury, or taken altogether, does not make out a case of bad faith, improper conduct, or improper motives." These observations adopted by Lord Justice Turner, preclude the necessity for further comment on the particular point to which they referred.

We have, in our criticism on this case, been obliged to reopen many questions, and refer to many facts which we would willingly commit to oblivion. The investigation has been productive of pain to all who were parties to it; let us, whatever our opinions on the matter may be, at least indulge the hope that it will eventuate in good.

